# Supplementary material for: Efficient magnetic switching in a correlated spin glass
Source: Nat Commun. 2023 Oct 2;14:6127. doi: 10.1038/s41467-023-41718-4 (PMC10543544; doi:10.1038/s41467-023-41718-4)
Supplement: Supplementary file 1 — Supplementary information [file 41467_2023_41718_MOESM1_ESM.pdf]

Supplementary information: Efficient magnetic switching in a correlated spin glass

Juraj Krempaský,[1,*] Gunther Springholz,[2] Sunil Wilfred D'Souza,[3] Ondřej Caha,[4] Martin Gmitra,[5,6] Andreas Ney,[2] C.A.F. Vaz,[1] Cinthia Piamonteze,[1] Mauro Fanciulli,[7] Dominik Kriegner,[8,9] Jonas A. Krieger,[10,†] Thomas Prokscha,[10] Zaher Salman,[10] Jan Minár,[3,‡] and J. Hugo Dil[1,11,§]

[1]*Photon Science Division, Paul Scherrer Institut, CH-5232 Villigen, Switzerland*

[2]*Institut für Halbleiter-und Festkörperphysik, Johannes Kepler Universität, A-4040 Linz, Austria*

[3]*New Technologies-Research Center University of West Bohemia, Plzeň, Czech Republic*

[4]*Dept. of Condensed Matter Physics, Masaryk University, Kotlářská 267/2, 61137 Brno ,Czech Republic*

[5]*Institute of Physics, P. J. Šafárik University in Košice, Park Angelinum 9, 040 01 Košice, Slovakia*

[6]*Institute of Experimental Physics, Slovak Academy of Sciences, Watsonova 47, 040 01 Košice, Slovakia*

[7]*LPMS, Université de Cergy-Pontoise, 95031 Cergy-Pontoise, France*

[8]*Institute of Physics ASCR, v.v.i., Cukrovarnická 10, 162 53 Praha 6, Czech Republic*

[9]*Dept. of Condensed Matter Physics, Charles University, Ke Karlovu 5, 121 16 Praha 2, Czech Republic*

[10]*Laboratory for Muon Spin Spectroscopy, Paul Scherrer Institute, CH-5232 Villigen PSI, Switzerland*

[11]*Institut de Physique, École Polytechnique Fédérale de Lausanne, CH-1015 Lausanne, Switzerland*

[*] juraj.krempasky@psi.ch

[†] Current address: Max Planck Institut für Mikrostrukturphysik, Weinberg 2, 06120 Halle, Germany

[‡] jminar@ntc.zcu.cz

[§] hugo.dil@epfl.ch

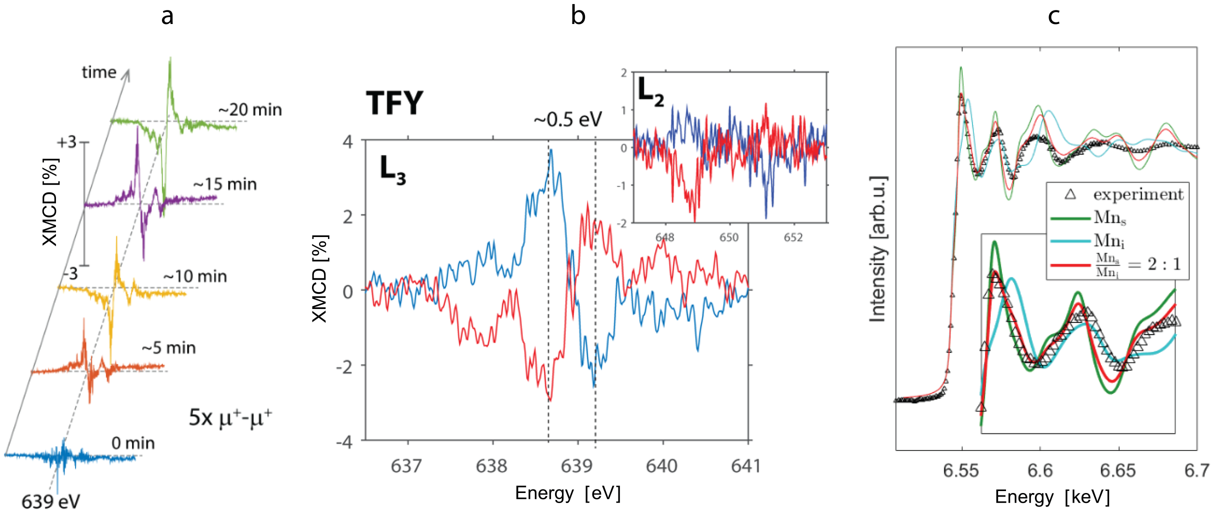

FIG. 1. Additional XMCD switch data and NEXAFS data. (a) Stacked plot of $\mathrm{Mn_{L3}}$ XMCD spectra measured with same circular light polarization μ^{+} under MSR-on regime. A change in x-ray helicity relative to the fixed magnetization direction is equivalent to a change in the magnetization direction relative to the fixed helicity. (b) Spontaneous switching in consecutive XMCD datasets recorded in total fluorescence yield (TFY) at T=5 K and B=0 T, zoomed at the $\mathrm{Mn_{L3}}$ and $\mathrm{Mn_{L2}}$ absorption edges. (c) NEXAFS Mn-K edge data fits. Panel inset shows the best fit obtained with $\mathrm{Mn_s}$:$\mathrm{Mn_i}$ occupancy close to 2:1.

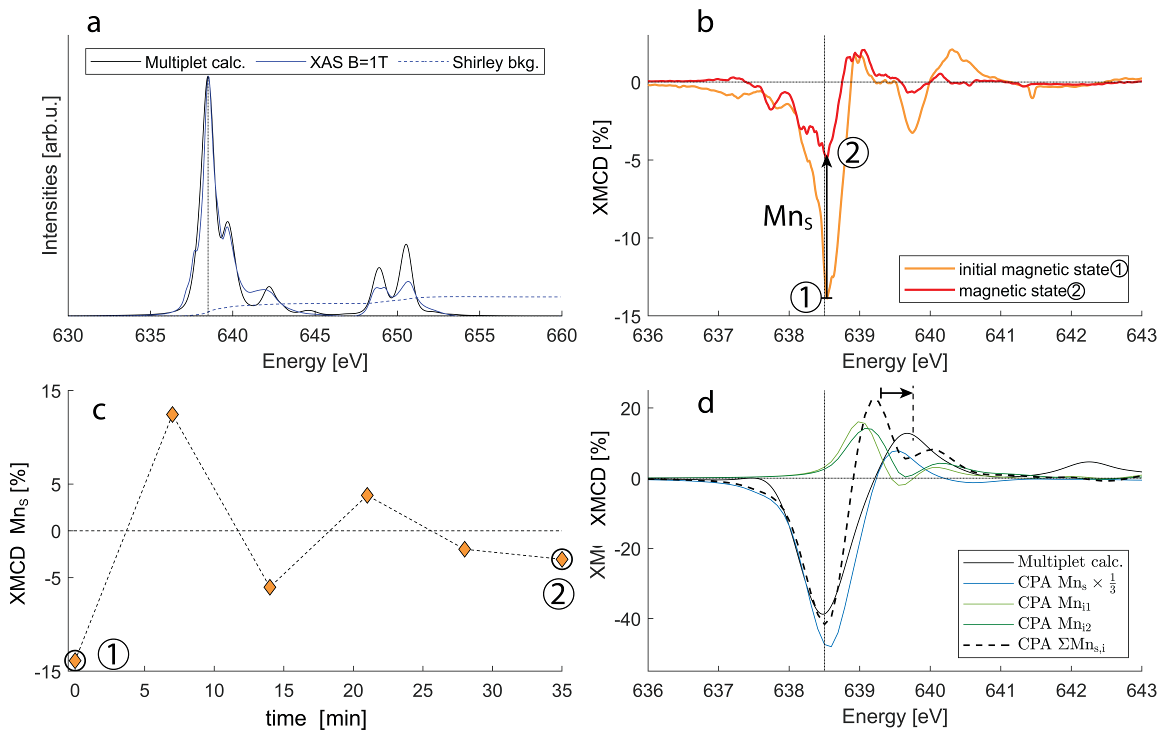

FIG. 2. Reduction of average magnetic moment on $\mathrm{Mn_s}$ and comparison to multiplet calculations. (a) Background subtracted XAS spectra measured at 6 T (10 K), compared with multiplet calculations. (b) $\mathrm{Mn_{L3}}$ XMCD measured at 10 K after FH-FC at 6 T, with two $\mathrm{Mn_s}$ states indicated as ① and ②, respectively. The reduction of $\mathrm{Mn_s}$ amplitude is due to several switching events whereby some of the $\mathrm{Mn_s}$ become part of the PM spin glass background. (c) Time dependence of the dichroism effects under MSR-on regime at $\mathrm{Mn_{L3}}$-edge between the initial magnetic state ① after FH-FC and magnetic state ② measured in consecutive XMCD measurements. After ≈35 min the magnetic interactions stabilize with dichroic signal typical for the MSR-regime, also seen in Fig. 1c and 4c. (d) Calculated XMCD spectra using CPA and multiplet calculations. For CPA approach the spectral contributions from individual Mn atoms are indicated. CPA is in good agreement with the experimental data, whereas the multiplet calculations show a clear shift in the spectral features, as indicated by the horizontal arrow.

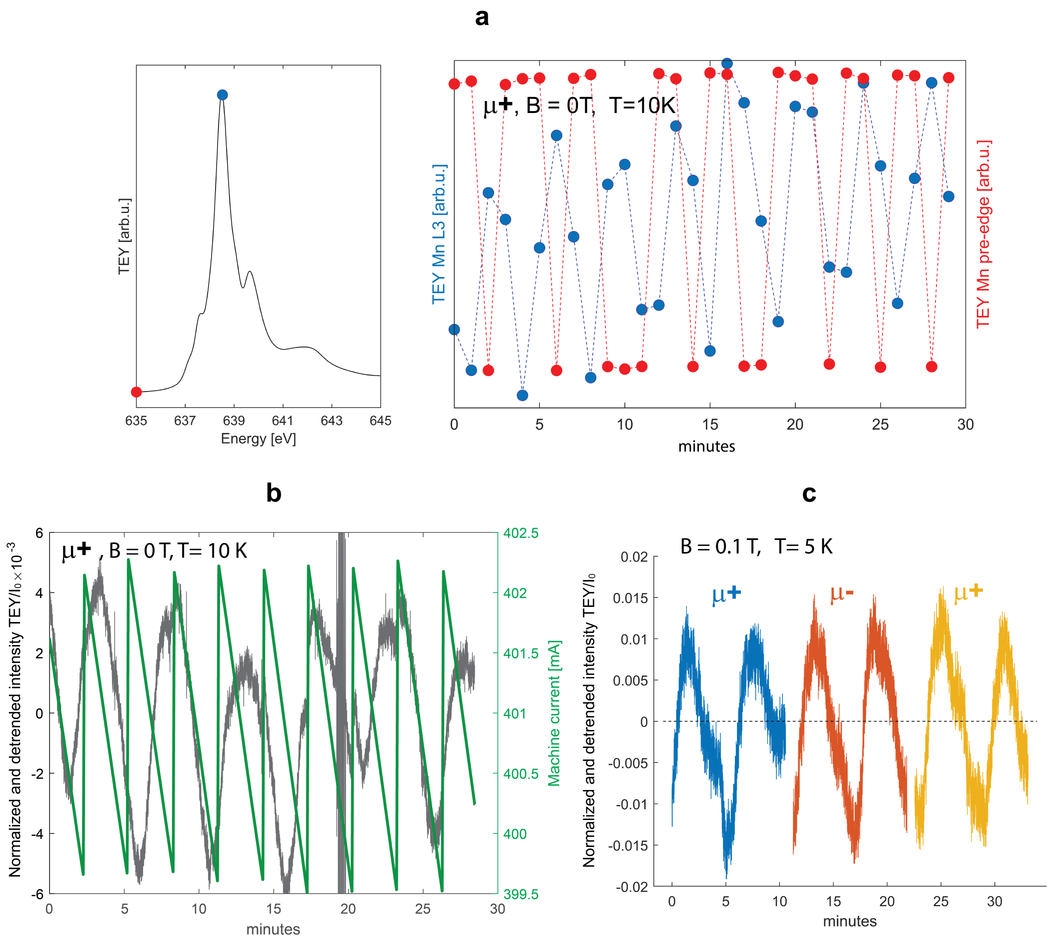

FIG. 3. Oscillations of TEY current under different conditions. (a) TEY time survey of $\mathrm{Mn_{L3}}$ pre-edge (red markers) and edge (blue markers) obtained during XMCD acquisition. (b) TEY time survey at $h\nu = 638.8$eV ($\mathrm{Mn_{L3}}$ edge) overlaid with the machine top-up current. (c) Series of TEY time surveys at $h\nu = 638.8$eV under applied B-field and different x-ray polarizations.

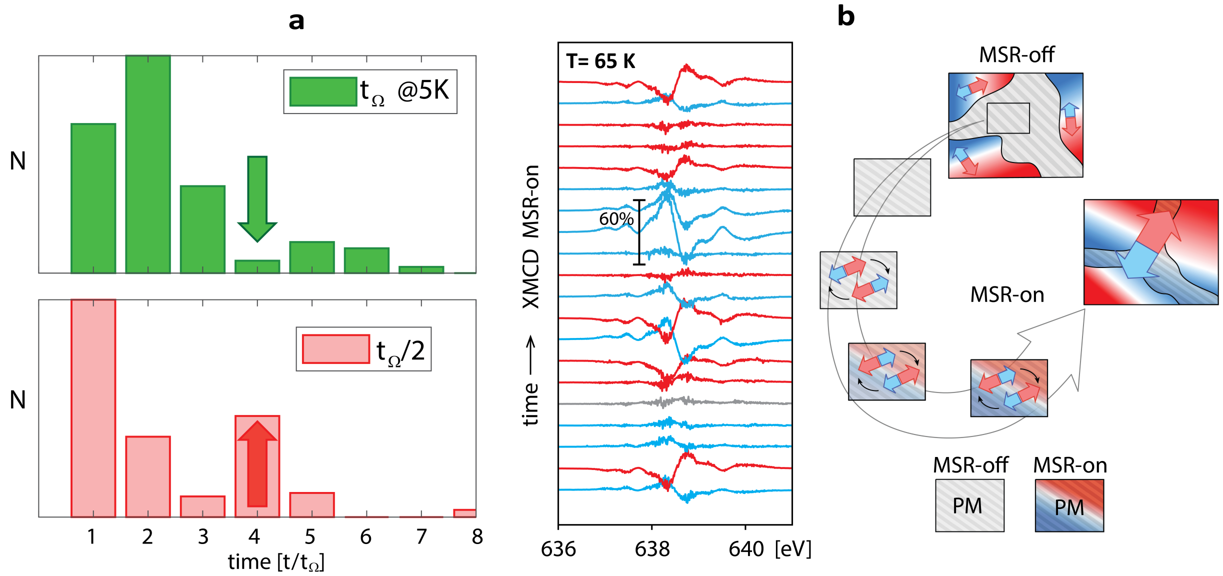

FIG. 4. Magnetic switching observed at higher temperature. (a) (top) Magnetostochastic switching residence time distribution $N(t)$ for a data set measured at 5 K, ZFC and t_Ω periodic drive obtained by measuring full XMCD spectra covering both $\mathrm{Mn_{L3}}$+$\mathrm{Mn_{L2}}$; compared with all data measured after FH-FC for 5, 45 and 65 K (red markers in Fig. 6b) where the switching statistics was evaluated from shorter energy scans across the $\mathrm{Mn_{L3}}$ only, i.e. by halving the periodic drive ($\frac{1}{2}t_\Omega$). The arrows highlight the $N(t)$ phase shift due to different stochastic resonance conditions [32]. (b) Series of $\mathrm{Mn_{L3}}$ XMCD spectra measured at 65 K, with maximum dichroism effect close to 60%. The pictographs illustrate enhancement of the FiM order (red-blue arrows) spreading across the magnetic clusters along with self-induced magnetization of the PM sea under MSR FiM switching.
